# Spatiotemporal evolution of pyroptosis and canonical inflammasome pathway in hSOD1^G93A^ ALS mouse model

**DOI:** 10.1186/s12868-022-00733-9

**Published:** 2022-08-09

**Authors:** Haoyun Zhang, Hao Li, Bingkun Huang, Shaoye Wang, Ying Gao, Fandi Meng, Yanchun Chen, Fenghua Zhou, Yingjun Guan, Xin Wang

**Affiliations:** 1 School of Basic Medical Sciences, Weifang Medical University, No. 7166 Baotong West Street, Weifang, 261053 Shandong China; 2 Neurologic Disorders and Regenerative Repair Lab of Shandong Higher Education, Weifang Medical University, No. 7166 Baotong West Street, Weifang, 261053 Shandong China; 3 School of Life Science and Technology, Weifang Medical University, No.7166 Baotong West Street, Weifang, 261053, Shandong China; 4 Department of Neurosurgery, Brigham and Women’s Hospital, Harvard Medical School, 221 Longwood Ave, Boston, MA 02115 USA

**Keywords:** Amyotrophic lateral sclerosis, Pyroptosis, Inflammasome, Neuroinflammation, Neurodegeneration

## Abstract

**Background:**

Evidences indicate that inflammasome compounds participate in amyotrophic lateral sclerosis (ALS), a fatal progressive motoneuron degenerative disease. Researchers have observed the expressions of nucleotide oligomerization domain (NOD)-like receptor protein 3 (NLRP3) related inflammasome components in specific regions of the central nervous system in different ALS models, but the cellular spatiotemporal evolution of this canonical inflammasome pathway and pyroptosis during ALS progression are unclear.

**Methods:**

The spinal cords of hSOD1^G93A^ mice (ALS mice) and age-matched littermates (CON mice) were dissected at pre-symptomatic stage (60 d), early- symptomatic stage (95 d), symptomatic stage (108 d) and late-symptomatic stage (122 d) of the disease. By using Nissl staining, double immunofluorescence labelling, qRT-PCR or western blot, we detected morphology change and the expression, cellular location of GSDMD, NLRP3, caspase-1 and IL-1β in the ventral horn of lumbar spinal cords over the course of disease.

**Results:**

Neural morphology changes and GSDMD^+^/NeuN^+^ double positive cells were observed in ventral horn from ALS mice even at 60 d of age, even though there were no changes of *GSDMD* mRNA and protein expressions at this stage compared with CON mice. With disease progression, compared with age-matched CON mice, increased expressions of GSDMD, NLRP3, activated caspase-1 and IL-1β were detected. Double immunofluorescence labeling revealed that NLRP3, caspase-1, IL-1β positive signals mainly localized in ventral horn neurons at pre- and early-symptomatic stages. From symptomatic stage to late-symptomatic stage, robust positive signals were co-expressed in reactive astrocytes and microglia.

**Conclusions:**

Early activation of the canonical NLRP3 inflammasome induced pyroptosis in ventral horn neurons, which may participate in motor neuron degeneration and initiate neuroinflammatory processes during ALS progression.

**Supplementary Information:**

The online version contains supplementary material available at 10.1186/s12868-022-00733-9.

## Background

Amyotrophic lateral sclerosis (ALS) is a common adult-onset progressive motor neuron (MN) disease characterized by degeneration of upper and lower MNs in the motor cortex, brain stem, and spinal cord [1–3], leading to muscle weakness and atrophy followed by paralysis. The majority of ALS patients die within 3–5 years after the onset of symptoms. Most ALS cases (~ 90% of all cases) are sporadic (sALS) with no obvious related risk factor and only 10% account for familial forms – familial ALS (fALS). It was reported that about 20% of fALS and 2% of all ALS cases are inherited with mutations in Cu^2+^/Zn^2+^ superoxide dismutase (SOD1) gene [4, 5]. Although the etiology of most ALS cases remains unknown, both genetic and environmental factors play a role in increasing the risk of developing ALS. Using hSOD1^G93A^ transgenic mice, Beers DR et al. suggested a paracrine mechanism of neuronal degeneration, in which pro-inflammatory and other toxic factors and/or neurotrophic factors (NTFs) deficiency may trigger motor neuron loss [6].

Pyroptosis (fiery death) is a pro-inflammatory form of cell death featured by early plasma membrane rupture dependent on gasdermin-D (GSDMD) proteins as an executioner of cell death [7]. After activation, the N-terminus of GSDMD accumulates on cellular membranes to form membrane pores, which leads to cell swelling and lysis [8]. GSDMD can be cleaved by activated caspase-1, it referred to as the canonical inflammasome pathway [9]. Otherwise, murine caspase-11 and human caspase-4/5 serve as cytosolic sensors in response to cytosolic lipopolysaccharide (LPS) to cleave GSDMD, which mediate noncanonical inflammasome pathway contributing to the clearance of cytosolic bacterial pathogens in vivo [10]. Pyroptosis plays an important role in multiple neurological diseases, such as multiple sclerosis, Alzheimer’s disease (AD), cerebral ischemia, and traumatic brain injury (TBI) [11, 12]. The role of pyroptosis in ALS is poorly characterized.

Inflammasomes are multiprotein complexes that function as intracellular sensors of environmental and cellular stress [13]. The nucleotide oligomerization domain (NOD)-like receptor protein 3 (NLRP3) inflammasome is well studied. Upon activation, NLRP3 assembles with the signalling adapter called apoptosis-associated speck-like protein (ASC) to cleave and active caspase-1 [14]. This NLRP3-caspase-1-mediated canonical inflammasome pathway plays an important role in innate immune reaction, which recognizes and responds to danger signals, such as altered host molecules, including misfolded proteins. Using the ALS^G93A^ mouse model, researchers suggested that aggregated SOD1 led to activation of canonical inflammasome [15–17]. Activated caspase-1 mediates proteolytic maturation and release of interleukin-1β (IL-1β) and IL-18, participating in inflammatory responses [18]. Elevated IL-1β levels and activated caspase-1 have been shown in the central nervous system (CNS) of mutant SOD1 transgenic mice and ALS patients [15].

Given the role of the inflammasome in ALS pathogenesis, in the present study, we evaluated the involvement of the pyroptosis in early MN degeneration of lumbar spinal cord. We also investigated the temporal expression and cell-type specific distribution of NLRP3 inflammasome components with disease progression in hSOD1^G93A^ mice. We found that the expression of inflammasome compounds were firstly observed in ventral horn neurons at pre-symptomatic stage of ALS mice.

## Materials and methods

### Ethical statement

All animal experiments were performed according to the National Research Council Guide for the Care and Use of Laboratory Animals and approved by the Animal Ethics Committee of Weifang Medical University (Date: 2018.02.26/2018-NO.156). This study was carried out in compliance with the ARRIVE guidelines.

### Animals

B6SJLF1/J mice and B6SJL-Tg (SOD1*G93A) 1Gur/J transgenic mice (hSOD1^G93A^ mice, ALS mice) were purchased from Jackson Laboratories (Bar Harbor, ME, USA). Male ALS mice were crossed with female B6SJLF1/J mice to produce ALS mice and non-transgenic littermate control mice (CON mice). To genotype animals, PCR using genomic DNA from tail clips was performed as suggested by the Jackson Laboratory. ALS mice (n = 44) and age-matched CON mice (n = 44) were randomly assigned into 4 groups: pre-symptomatic stage (60 d), early-symptomatic stage (95 d), symptomatic stage (108 d), and late-symptomatic stage (122 d). All mice were housed in the animal care facility at 22 ± 1 °C and 50–70% humidity under a 12-h light–dark cycle, with ad libitum access to food and water.

### Nissl staining

After anaesthetization (1% pentobarbital sodium at 40–50 mg/kg, i.p.), the mice (n = 3/group/time point) were transcardially perfused with 4% paraformaldehyde in 0.1 M phosphate-buffered saline (PBS, pH 7.4), and spinal cords were post-fixed overnight with 4% PFA at 4 °C. The lumbar enlargement spinal cord was sliced in 7 μm sections using a Leica CM3050S cryostat. After stained by Nissl staining Solution (Sangon Biotech, Shanghai, China) for 30 min at room temperature, the slices were dehydrated in gradient alcohol and cleared in xylene. Sample slides were observed and photographed with an Olympus BX53F microscope (Tokyo, Japan).

### Double immunofluorescence labelling

After blocked with 10% normal goat serum (containing 0.3% Triton X-100), sections (prepared as above) were incubated overnight at 4 °C with combined primary antibodies simultaneously: mouse /rabbit anti-NeuN (1:200, MA5-33,103, Invitrogen, USA or 1:100, 24,307, Cell Signaling Technology, USA), mouse/rabbit anti-GFAP (1:500, MA1-35,377, Invitrogen or 1:200, BM4287, Boster, China) and mouse/rabbit anti-Iba1 (1:100, 012–26,723/019–19,741, Wako, Japan) with rabbit anti-NLRP3 (1:200, bs-6655R, Bioss, China), rabbit anti-caspase-1(1:100, 22,915–1-AP, Proteintech), mouse anti-GSDMDC1 (1:100, sc-393656, Santa Cruz, USA) and rabbit anti-IL-1β (1:200, ab283818, Abcam, USA). All primary antibodies were diluted in 0.01 M PBS (pH 7.4) containing 1% BSA. Sections were washed 3 × 10 min with PBS prior to incubation with appropriate conjugated secondary antibodies: goat anti-mouse IgG H&L/FITC (bs-0296G-FITC, Bioss) and goat anti-rabbit IgG H&L/Alexa Fluor 594 (bs-0294P-AF594, Bioss) or goat anti-rabbit IgG H&L/FITC (bs-0295G-FITC) and goat anti-mouse IgG H&L/Alexa Fluor 594 (bs-0296G-AF594, 1:200, Bioss) at room temperature in dark. Cell nuclei were counterstained with Hoechst 33,258 (Invitrogen). All sections were mounted with an anti-fading medium (Solarbio, Beijing, China). Sections were examined using an Olympus BX53F microscope (Tokyo, Japan). Control slices incubated in a solution without primary antibodies to give a measure of nonspecific background staining.

### Quantitative real-time PCR (qRT-PCR) analysis

The spinal cord (n = 4/group/time point) was forced out of the spinal column using 0.01 M PBS with a 10 ml syringe. Total RNA was isolated from the spinal cord of CON mice and ALS mice using the Trizol Reagent (Invitrogen) according to the manufacturer’s instructions. Then, 2 μg of total RNA was reversely transcribed in a 20 μl reaction with oligo-dT primers using a reverse-transcription system (Evo M-MLV RT Kit with gDNA Clean for qPCR, Accurate Biology, China). qRT-PCR was performed to determine the expression of *GSDMD*, *NLRP3*, *caspase-1 and IL-1β*. The following primers were employed (Table 1). No reverse transcriptase control, and water as no template control were used as negative controls.

**Table 1:** List of Primers

Primer	Sequence (5′to 3′)
*GSDMD*	sense: GGTGCTTGACTCTGGAGAACTG
	antisense: GCTGCTTTGACAGCACCGTTGT
*NLRP3*	sense: CCTGGGGGACTTTGGAATCAG
	antisense: ATCCTGACAACACGCGGA
*Caspase-1*	sense: CCCCAGGCAAGCCAAATC
	antisense: TTGAGGGTCCCAGTCAGTCC
*IL-1β*	sense: GGGCTGGACTGTTTCTAATGC
	antisense: GGTTTCTTGTGACCCTGAGC
*GAPDH*	sense: CCCCCAATGTATCCGTTGTG
	antisense: GTAGCCCAGGATGCCCTTTAGT

Amplification and detection were performed in a standard tube using the Bio-Rad CFX96 Detection System (BioRad, CA, USA), with the following conditions: an initial hold at 95 °C for 30 s, followed by 40 cycles at 95 °C for 5 s and 60 °C for 45 s. The relative expression level of each mRNA was calculated using the ^ΔΔ^Ct method normalizing to *GAPDH* and relative to the control samples.

### Western blot

The spinal cords (n = 4/group/time point) were obtained as described above. Protein extraction was performed on ice using ice-cold reagents. The spinal cords were lysed with RIPA lysis buffer (Beyotime Biotechnology, Beijing, China). Insoluble material was removed by centrifugation. A total of 40–60 μg of protein was separated using 10% or 12% SDS-PAGE and then transferred onto PVDF membranes. Membranes were blocked in 10% non-fat milk for 1 h. For NLRP3, the membranes were cut into proper bands following by incubated with rabbit anti-NLRP3 and mouse anti-GAPDH primary antibodies. For caspase-1, after blotted for caspase-1, the membrane were cut from about 40kD to 35kD to hybrid with primary antibody of mouse anti-GAPDH. The membranes were incubated with the following antibodies: mouse anti-GSDMDC1 (1:1000, sc-393656, Santa Cruz), rabbit anti-NLRP3 (1:1000, bs-6655R, Bioss), rabbit anti-caspase-1(1:1000, 22,915–1-AP, Proteintech), rabbit anti-IL-1β (1:2000, ab283818, Abcam, USA) and mouse anti-GAPDH (1:5000, sc-365062, Santa Cruz) overnight at 4 °C. After three washes with TBST, membranes were then incubated in HRP-conjugated goat anti-rabbit or goat anti-mouse secondary antibody (1:5000, sc-2004/sc-2005, Santa Cruz) at room temperature for 1 h, and washed with TBST for 3 times. Cross-reactivity was visualized using ECL detection reagents and were quantified with ImageJ software. The results were normalized to GAPDH levels.

### Statistical analysis

All data were analysed with the statistical program GraphPad Prism 7 (Graphpad Software Inc., CA, USA). All values were presented as mean ± SEM. An unpaired two-tailed Student’s *t*-test was performed to analyse the differences between the ALS group and CON group at each time point. A *P* value of < 0.05 indicated statistical significance.

## Results

### Neurodegeneration in the ventral horn of ALS mice lumbar spinal cord

**Fig. 1:**
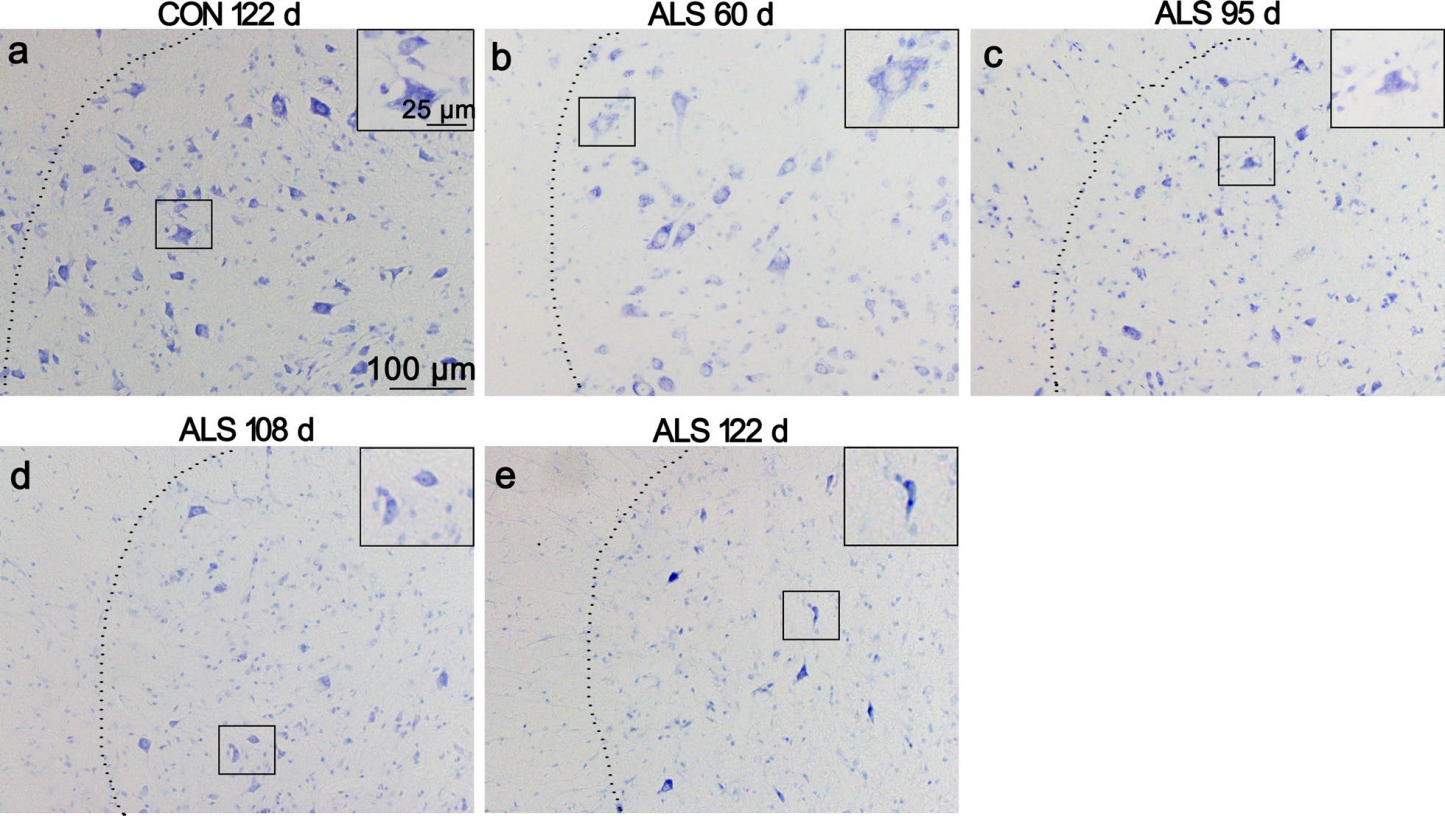
Neural degeneration in the ventral horn of ALS mice lumbar spinal cord. Representative photomicrographs of Nissl-stained lumbar sections of 122 d CON mice **a**, 60 d ALS mice **b**, 95 d ALS mice **c**, 108 d ALS mice **d** and 122 d ALS mice (**e**, scale bar 100 μm) are shown. Inserts depict higher magnifications of the overview images (Scale bar 25 μm). In ALS mice, altered neurons were frequently observed from 60 d of age with vacuolation in the soma. With disease progression, spongiform degeneration and loss of large motoneurons was detected.

Ngo’s study showed that the numbers of motor neurons in SOD1^G93A^ mice was significantly less compared with age-matched wild-type controls at all stages of disease, from pre-symptomatic age (30–36 d) to end-stage (150–180 d) [19]. Using Nissl staining, we also confirmed this neural pathological change. The normal healthy neurons in the ventral horn of CON mice's lumbar spinal cord showed many large Nissl bodies distributed in the soma (Fig. 1a). Whereas abnormal neurons in ALS mice were found as early as 60 d of age, with a white foamy cytoplasmic staining in the soma because of reduction in Nissl bodies and neuronal vacuolation (Fig. 1b). With disease progression, neuronal damage was much clear, reduced Nissl body, disintegrated and dark cytoplasm, and loss of a large portion of motor neurons from 95 to 122 d in lumbar sections (Fig. 1c-e).

### Increased expression and cell specific distribution of GSDMD in lumbar spinal cord with disease progression

**Fig. 2:**
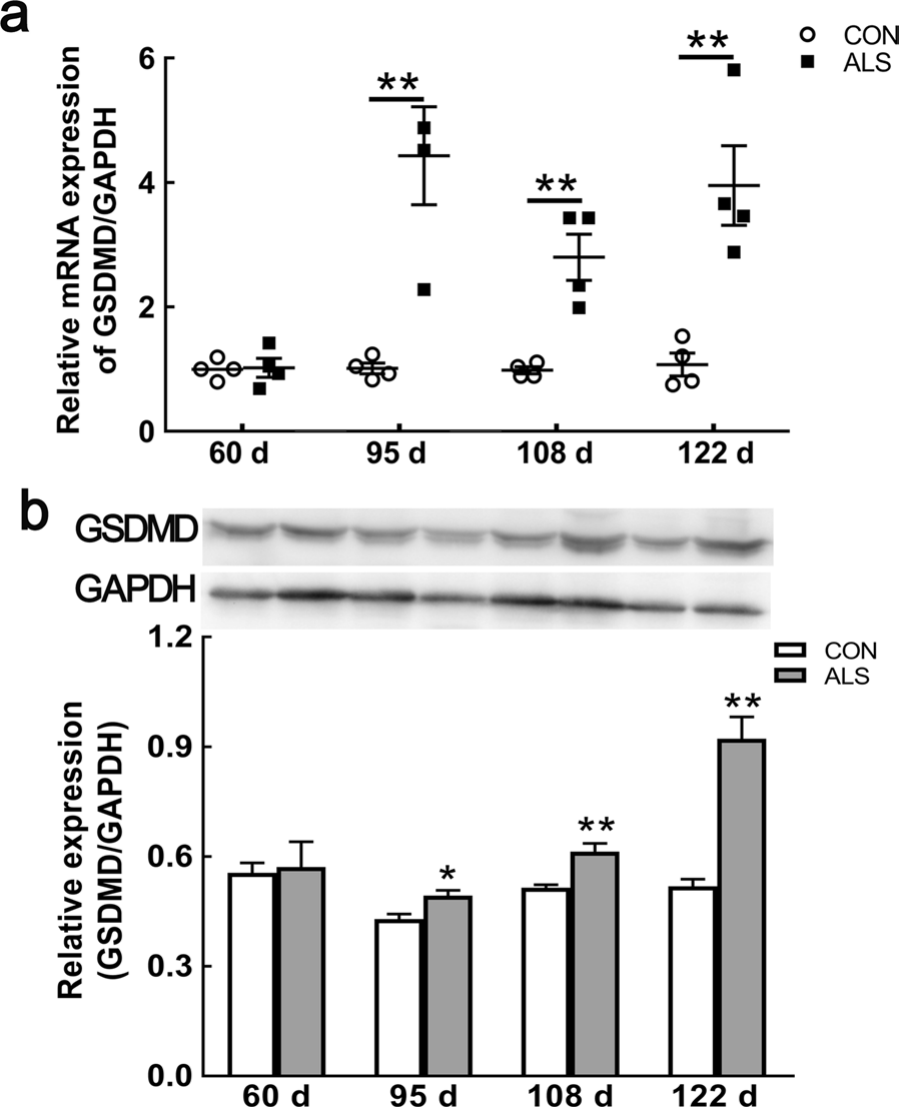
Upregulation of GSDMD in the spinal cord of ALS mice with disease progression. mRNA expression of *GSDMD* at 60 d, 95 d, 108 d and 122 d of ALS mice, compared with CON mice **a**. Protein expression of GSDMD at 60 d, 95 d, 108 d and 122 d in ALS mice **b**. Data represent means ± SEM (n = 4). ^*^P < 0.05, ^**^P < 0.01

**Fig. 3:**
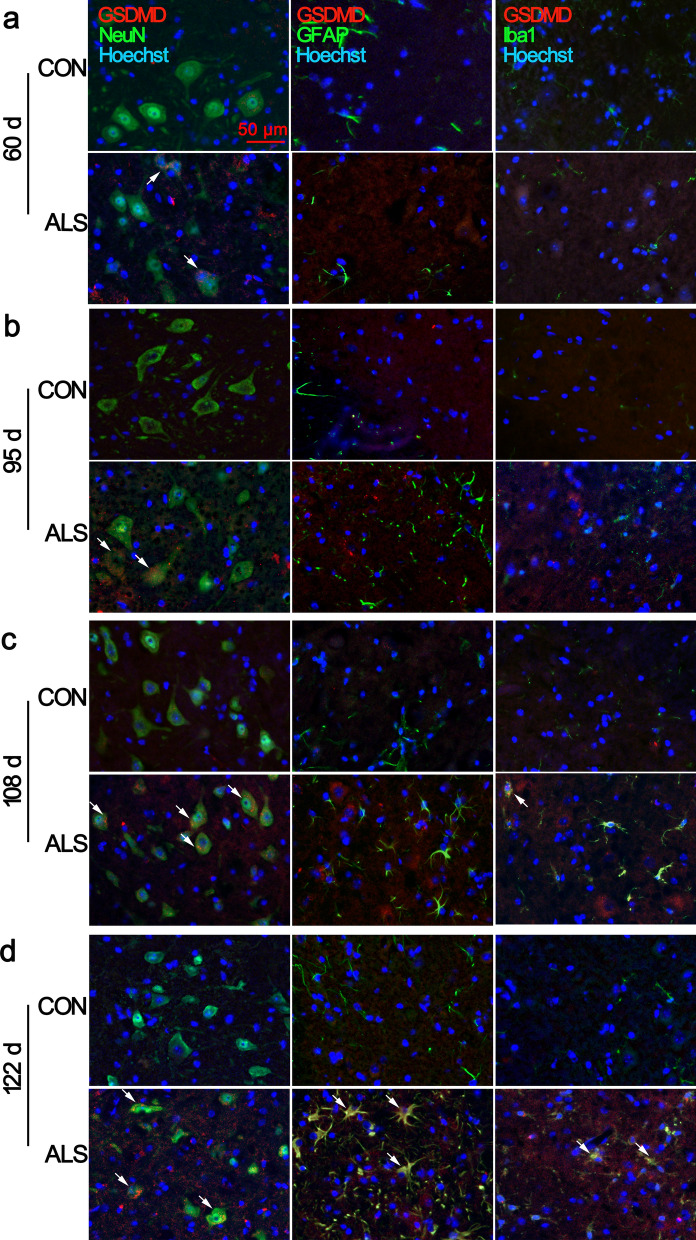
Increased expression of GSDMD in ventral horn neurons of ALS mice lumbar spinal cord. Double immunofluorescence staining showed that GSDMD (red) /NeuN (green) double-positive neurons were first observed at 60 d, 95 d of age in the ventral horn of ALS mice **a**, **b**. With disease progression, GSDMD positive signal was observe in glial cells at 108 d and 122 d of ALS mice **c**, **d**. Arrows indicate double-labelled cells. Scale bar = 50 μm

Pyroptosis is a type of programmed cell death, which is different from apoptosis causing membrane rupture by GSDMD N-terminal cleavage fragments. In ALS mice, from 95 to 122 d, the mRNA and protein expressions were significantly elevated compared with CON mice (Fig. 2a, b; Additional file 1: Fig. S1). We then examined the cell specific expression of GSDMD in the ventral horn of the lumbar spinal cord. As shown in Fig. 3, the increased immunoreactivity of GSDMD was initially detected in ALS mice at age of 60 d (Fig. 3a, arrows in the panels in the second row; Additional file 2: Fig. S2), and persistent at 95 d, and 108 d, which was primarily found in NeuN positive cells (Fig. 3b, c), in consistent with neurodegeneration. With disease progression, at 122 d, there were plenty of GSDMD/GFAP and GSDMD/Iba1 double positive cells (Fig. 3d; Additional file 2: Fig. S2). Very low expressions of GSDMD were observed in age-matched CON mice.

### Upregulation of NLRP3 inflammasome components in the spinal cord of ALS mice

**Fig. 4:**
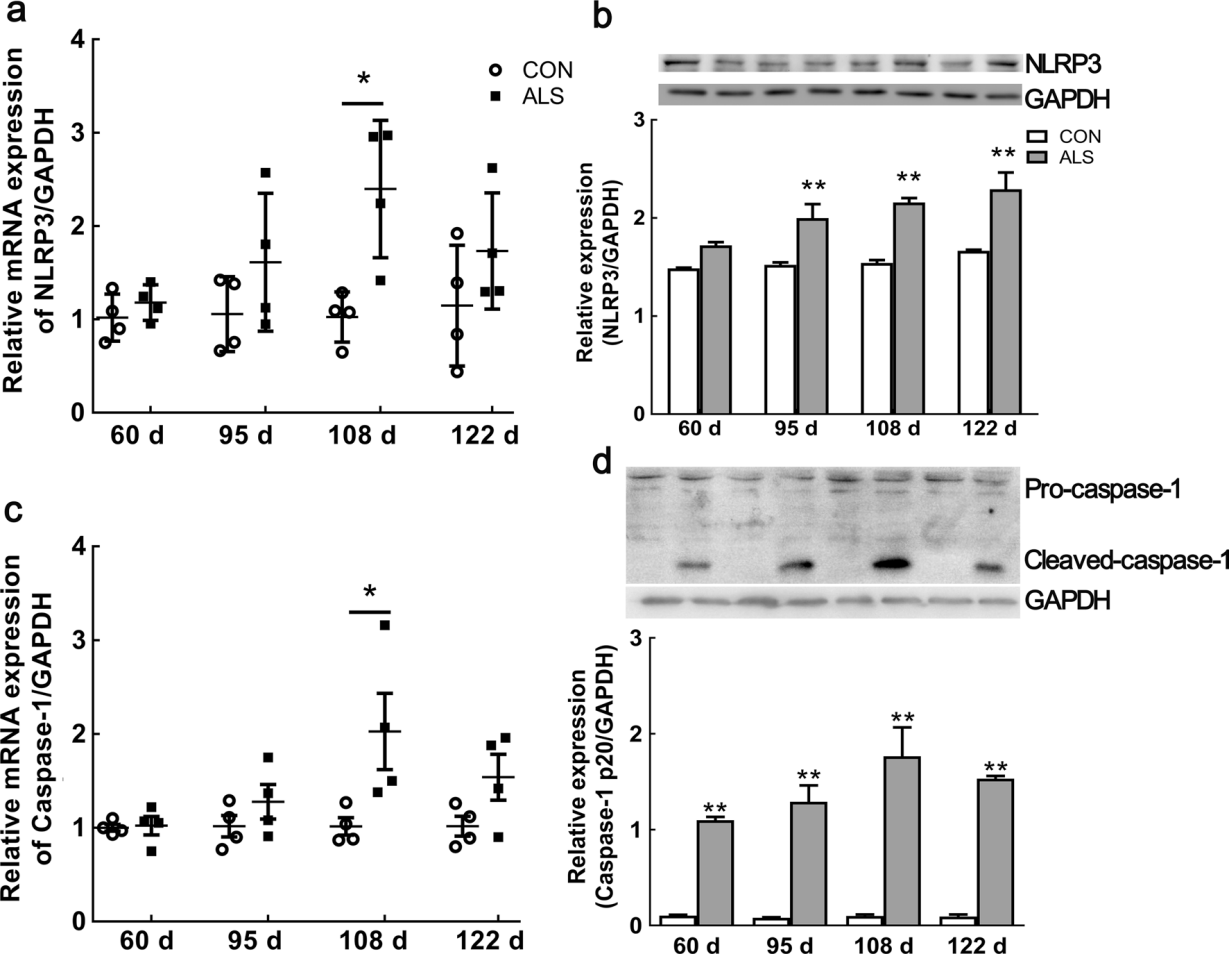
Upregulation of NLRP3 and activated caspase-1 in the spinal cord of ALS mice with disease progression. mRNA expression of *NLRP3* at 60 d, 95 d, 108 d and 122 d of ALS mice, compared with CON mice **a**. Protein expression of NLRP3 at 60 d, 95 d, 108 d and 122 d in ALS mice **b**. mRNA expression of *caspase-1* at 60 d, 95 d, 108 d and 122 d of ALS mice, compared with CON mice **c**. Caspase-1 activation from the pre-symptomatic stage to the end stage in ALS mice **d**. Data represent means ± SEM (n = 4). ^*^P < 0.05, ^**^P < 0.01

NLRP3 is crucial for subsequent activation of the other inflammasome components [13]. Using qRT-PCR, we investigated the dynamic expression of *NLRP3* mRNA and protein in the spinal cord during disease progression. Compared with age-matched CON mice, we found that the mRNA expression of *NLRP3* was increased at each time point in ALS mice, but only reached significant elevation at 108 d (~ 2.5-fold, *P* < 0.01; Fig. 4a). As shown in Fig. 4b, the NLRP3 protein was significantly increased at 95 d, 108 d, and 122 d by ~ 1.3, 1.4, and 1.4-fold, respectively (*P* < 0.01, Additional file 3: Fig S3, S4).

Assembled NLRP3 binds to ASC to induce caspase-1 activation. Previous study indicated caspase-1 activation in the ventral horn sections at the lower thoracic level of SOD1^G93A^ mice [20]. Using qRT-PCR analysis, we found the increase of *caspase-1* mRNA starting at 95 days of age, peaked at 108 days (~ 2-fold above CON mice; Fig. 4c). Western blot analysis showed caspase-1 p20 active fragment in ALS mice spinal cord at 60 d, and the caspase-1 cleavage product presented throughout the disease. Limited caspase-1 p20 active fragments were detected in non-transgenic littermates at any age (Fig. 4d; Additional file 4: Fig S5, S6). These results indicate early activation of NLRP3-caspase-1 inflammasome from pre-symptomatic.

### Early expression of NLRP3-caspase-1 inflammasome compounds in neurons from pre-symptomatic stage

**Fig. 5:**
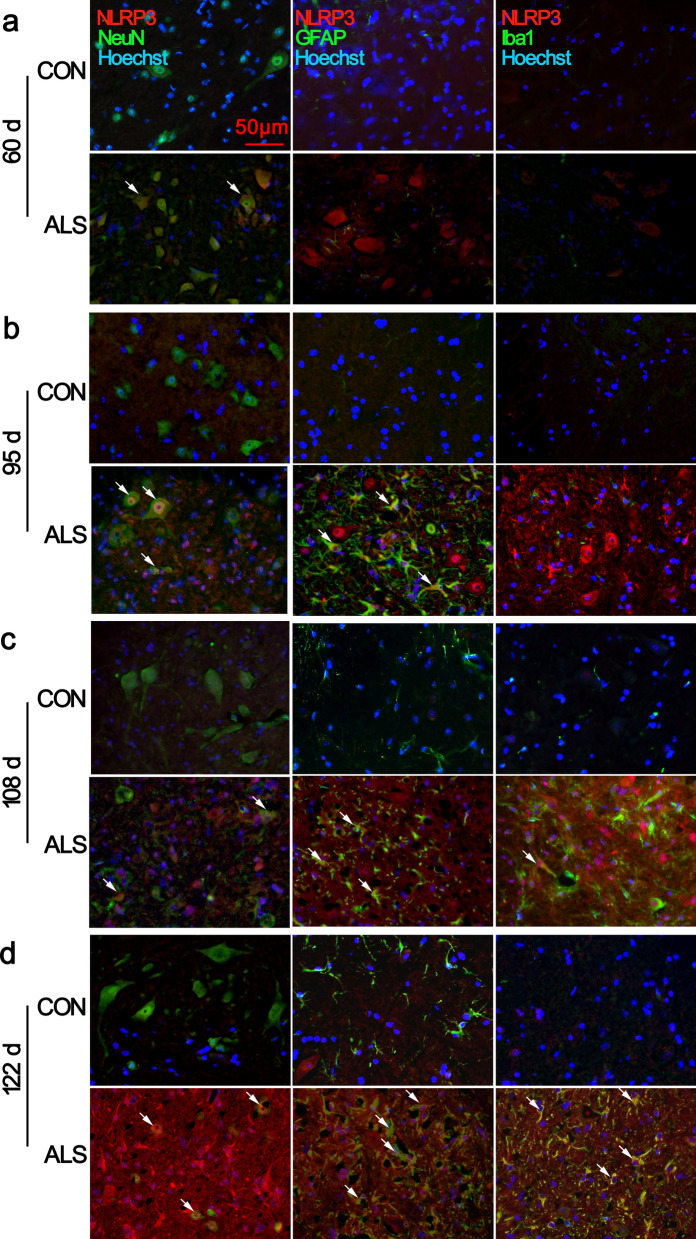
Cell-specific location of NLRP3 in the lumbar spinal cord. Representative images of double immunofluorescence staining for NLRP3 with NeuN, GFAP, or Iba1 are shown. At 60 d, NLRP3 was mainly expressed in the neuronal cytoplasm of ventral horn spinal cord of ALS mice, and low expression was found in CON mice **a**. Dramatic co-localization with neurons and activated GFAP^+^ astrocytes in ALS mice was observed at 95 d **b**. Increased NLRP3 expression was observed in GFAP^+^ and Iba1^+^ cells in the ALS spinal cord **c**, **d**. Arrows indicate double-labelled cells. Scale bar 50 μm

To better define the cell types expressing NLRP3, we performed double immunofluorescence staining for NLRP3 in combination with the neuron marker NeuN, astrocyte marker GFAP, and microglia marker Iba1. NLRP3 immunoreactivity was detected in a few cells in the CON mice at all timepoints (Fig. 5; Additional file 5: Fig. S7). The NLRP3-positive cells were found colocalized with NeuN cells in the ventral horn of the lumbar spinal cord of ALS mice at 60 d and 95 d (Fig. 5a, b, arrows in the panels in the second row). At 95 d with activation of astrocytes, we detected some NLRP3/GFAP double-positive cells (Fig. 5b, arrows). At 108 d and 122 d, in addition to remaining NeuN positive cells, robust NLRP3 positive signals co-stained with GFAP and Iba1 (Fig. 5c, d, arrows) were observed.

**Fig. 6:**
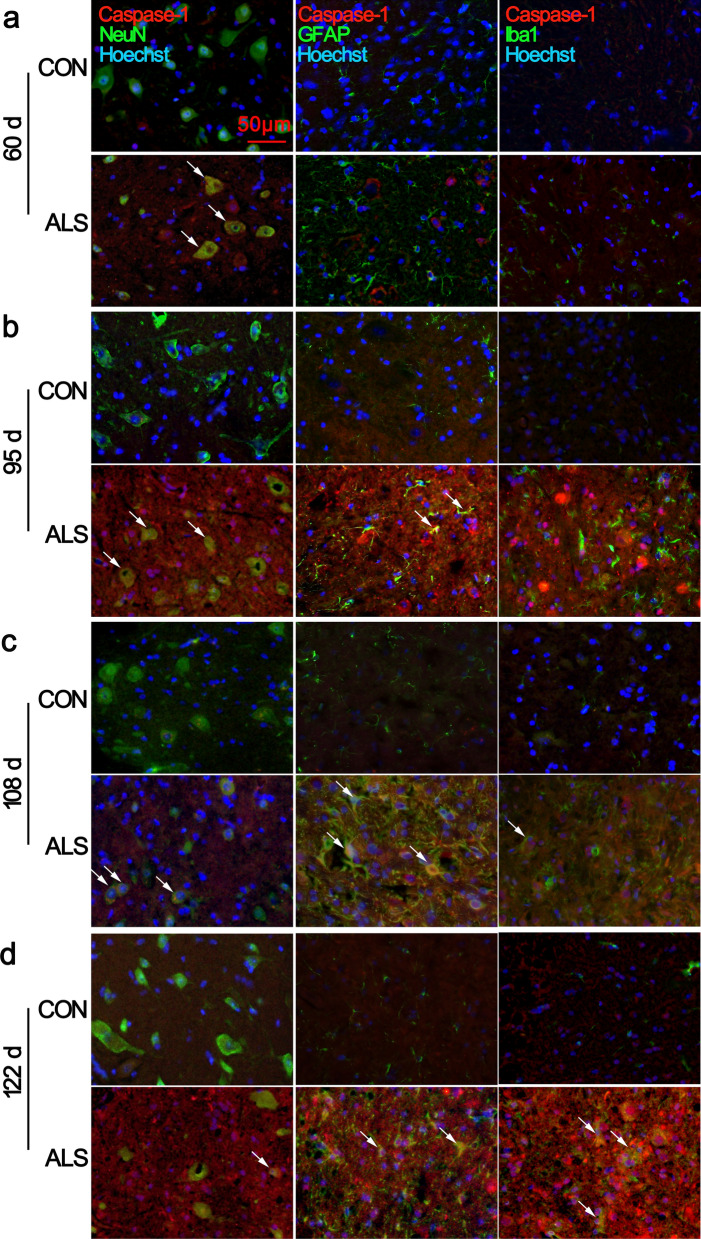
Cell-specific location of caspase-1 in the lumbar spinal cord. Representative images of double immunofluorescence staining for caspase-1 with NeuN, GFAP, or Iba1 are shown. At 60 d, caspase-1 was mainly expressed in the neuronal cytoplasm of ventral horn spinal cord of ALS mice, and low expression was found in CON mice **a**. Increased co-localization of caspase-1 with activated GFAP^+^ astrocytes and Iba1^+^ microglia in ALS mice were observed from 95 to 122 d **b**, **c**, **d**. Arrows indicate double-labelled cells. Scale bar 50 μm

Next double immunofluorescent analysis defined the identity of cells expressing caspase-1 in the lumbar spinal cords. At 60 d, the ventral horn of ALS mice maintained detectable caspase-1/NeuN double-positive cells (Fig. 6a, arrows in the panels in the second row. Additional file 6: Fig. S8), suggesting that caspase-1 is expressed predominantly in neurons in the pre-symptomatic stage. By the time of 95 d of age, when motor neurons begin to lose and the ALS mice are clinically affected, we detected that the caspase-1 positive inclusions were greatly increased in the ventral horn neurons and some reactive astrocytes (Fig. 6b, arrows). At 108 d and 122 d of age, more caspase-1-positive debris was present in activated astrocytes, microglia, and a few surviving neurons (Fig. 6c, d, arrows. Additional file 6: Fig. S8). In CON animals, caspase-1 positive signal could be barely detected at any time point.

### Increased expression of IL-1β in the ventral horn of ALS mice

**Fig. 7:**
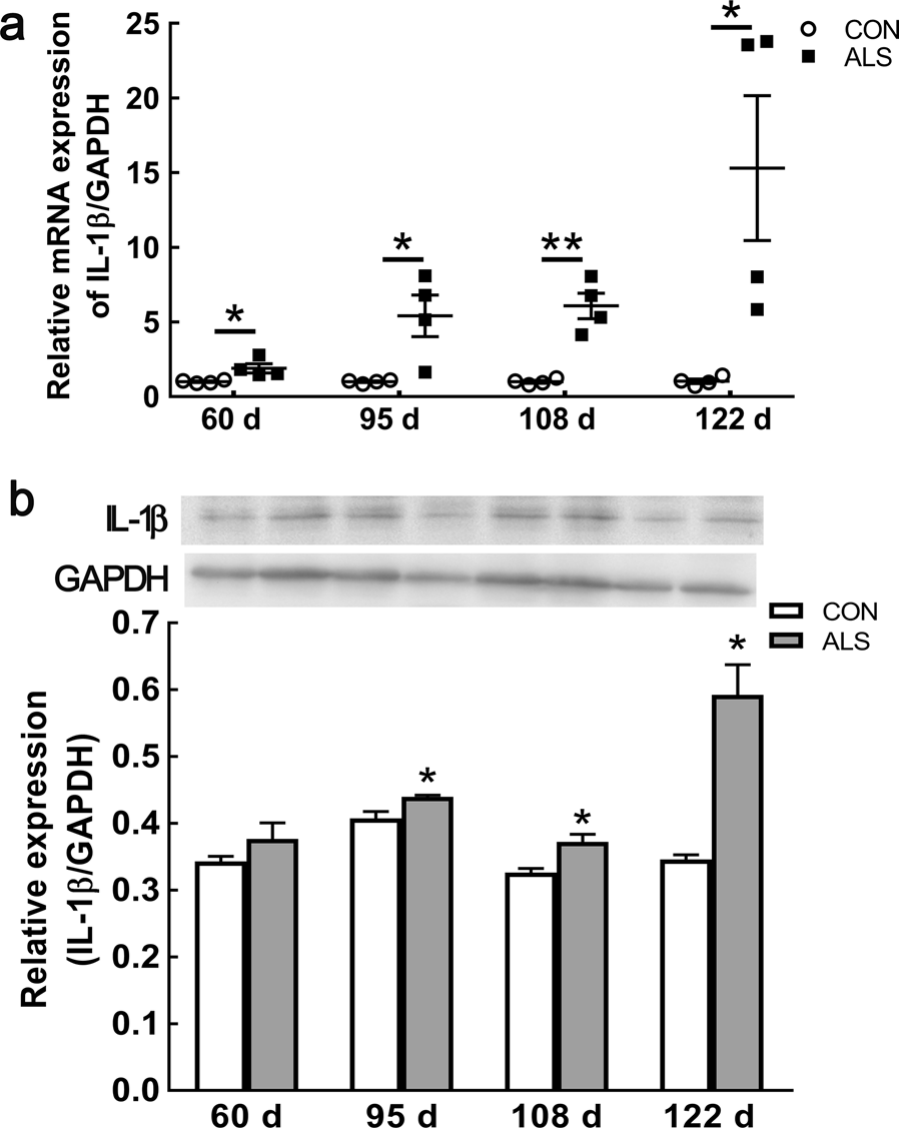
Upregulation of IL-1β in the spinal cord of ALS mice with disease progression. mRNA expression of *IL-1β* at 60 d, 95 d, 108 d and 122 d of ALS mice, compared with CON mice **a**. Protein expression of IL-1β at 60 d, 95 d, 108 d and 122 d in ALS mice **b**. Data represent means ± SEM (n = 4). ^*^P < 0.05, ^**^P < 0.01

Caspase-1 activation contributes to the mature of IL-1β and IL-18, and pyroptosis facilitates the release of these cytokines. IL-1β is increased in the ALS model and contributes to the disease’s progression [20, 21]. In the present study, we observed that compared with age matched CON mice, the increased expressions of* IL-1β* mRNA were detected in the ALS mice lumbar spinal cord from pre-symptom stage to late-symptom stage (Fig. 7a). Western blot indicated an increase tendency of IL-1β at 60 d, and significantly elevated from 95 to 122 d (Fig. 7b; Additional file 1: Fig. S1).

**Fig. 8:**
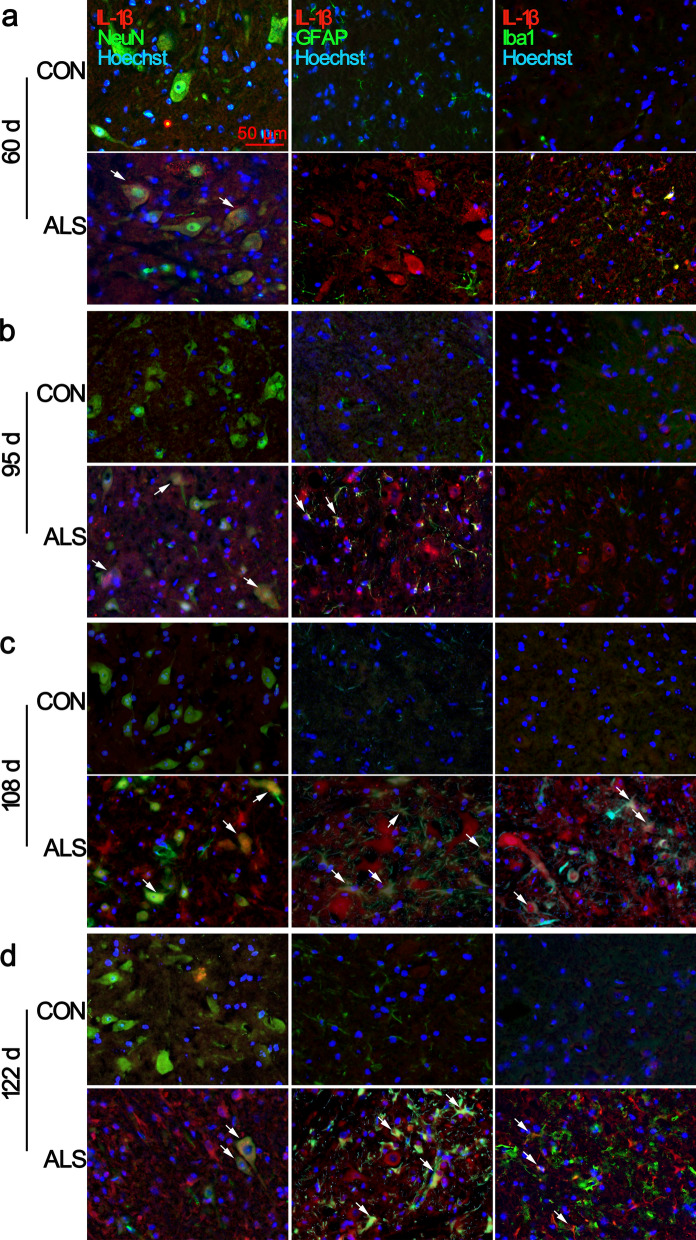
Cell specific location of IL-1β in the lumbar spinal cord. Representative images of double immunofluorescence staining for IL-1β with NeuN, GFAP, or Iba1 are shown. At 60 d, increased expression of IL-1β was observed in the neuronal cytoplasm of ventral horn spinal cord of ALS mice, and low expression was found in CON mice **a**. Besides NeuN^+^/IL-1β^+^ double positive cells, dramatic co-localization of IL-1β with activated GFAP^+^ astrocytes and Iba1^+^ microglia in transgenic ALS mice were observed from 95 to 122 d **b**, **c**, **d**. Arrows indicate double-labelled cells. Scale bar 50 μm

We focused on the distribution of IL-1β with ALS progression. The double immunofluorescent analysis showed that at age of 60 d in ALS mice, the density of IL-1β was slightly greater in the ventral horn of the lumbar spinal cord than that of their CON littermates. The positive signals were mainly localized in NeuN positive cells (Fig. 8a, arrows in the panels in the second row; Additional file 7: Fig. S9). At age of 95 d, the number of IL-1β/NeuN double-positive cells, as well as IL-1β^+^/GFAP^+^ and IL-1β^+^/Iba1^+^ cells were increased (Fig. 8b, arrows). As the disease progressed, there was a notable increase in IL-1β/GFAP and IL-1β/Iba1 double-labelling cells. Some residual neurons were also shown strong IL-1β positive signals in symptomatic and late-symptomatic stage mice (Fig. 8c–d, arrows; Additional file 7: Fig.S9).

## Discussion

Increasing evidence indicates that inflammasome-related neuroinflammation participates in ALS pathogenesis [22]. In the present study, we observed pyroptosis-related protein GSDMD were initially upregulated in the ventral horn neurons at the pre-symptomatic stage of ALS, in line with neural morphological changes. In contrast to CON mice, we found that inflammasome components, NLRP3, caspase-1, IL-1β were increased in the spinal cord of ALS mice, and firstly detected in lumbar ventral horn (Additional file 4) neurons over the course of ALS progression. These findings indicate that pyroptosis and related canonical inflammasome pathway participate in the early loss of spinal cord MNs, which may participate in further activation of glial cells to initiate chronic neuroinflammation.

The innate immune functions are generally helpful in keeping the host’s homeostasis by efficiently detecting and eliminating sterile tissue damage, metabolic alterations, and general stress in tissues. However, the overabundance of inflammasome activation can also lead to non-resolving inflammatory reactions [23]. In fALS cases, mutant SOD1 (mSOD1) seems to impair the protein degradation process through proteasome pathway and autophagy and causes misfolded proteins aggregation in the cell [24]. Persistent misfolded proteins in the CNS can activate NLRP3 inflammasome, which has emerged as a central neuroinflammatory mechanism that can drive neurodegeneration [18, 25]. Gugliandolo et al. [16] observed the increase of NLRP3 inflammasome components and activation of caspase-1 in the brain of SOD1^G93A^ rats at the end stage of disease, which in turn led to the amplification of IL-18 and IL-1β release. Kadhim and colleagues [26] reported a robust in-situ expression of IL-18 and activated NLRP3-caspase-1 inflammasome in sALS brains. They hypothesized a “vicious cycle” whereby cytokine-induced neural cell injury/death could significantly contribute to disease progression. Several studies, using mice of the same strain, revealed that NLRP3 associated with ASC were significantly up-regulated in the anterodorsal thalamic nucleus at pre- and early-symptomatic stage of ALS [17]. Our study presents evidence that NLRP3 upregulation and caspase-1 activation are accompanied by motor neurons neurodegeneration in the spinal cord in line with recent studies [27, 28].

Pyroptosis is an inflammation-associated cell death that is mediated by inflammasome and subsequent caspase-1 activation [29, 30]. In neurological degenerative disorders, such as Parkinson's disease and AD, several studies highlight the role of neuronal cell pyroptosis to be a contributor to disease progression [31–33]. In this study, we found GSDMD expression in ventral horn neurons from the pre-symptomatic stage, suggesting (Additional file 5) that pyroptotic neuronal death plays a role in MN loss, and aggravating neuroinflammation in ALS. Our data demonstrated that at 122 d, GSDMD immunolabelling was also expressed within GFAP^+^ cells and Iba-1^+^ cells, in this stage, we detected obvious spinal cord atrophy.

Regarding neuroinflammation, there are a lot of studies focusing on the activation of glial cells, so-called “gliosis”. Johann et al. found that from 14 w of age, the NLRP3 is mainly expressed in astrocytes in the spinal cord of ALS mice. However, they reported morphologically abnormal motoneurons with vacuolation in the spinal cord of SOD1 mice from pre-symptomatic (9 W), and a 30% loss of ChAT^+^ neurons already at 9 W which proceeded to 50% loss at 14 W [28], indicating motor neurons degeneration is earlier than gliosis. We want to verify the cell-specific location of inflammasome from disease early stage. NLRP3 compounds (Additional file 6) are expressed not only in classical immune cell microglia but also in non-immune cells such as neuron, astrocyte in CNS [34]. Using double immunofluorescence labelling, we observed inflammasome components, such as NLRP3, caspase-1 and IL-1β, mainly localized in ventral horn neurons of lumbar spinal cord as early as pre-symptomatic stage (60 d). As the disease progressed, inflammasome components co-labelled with activated astrocytes and microglia were sharply increasing at 108 d and 122 d. In combination with these evidence, our findings suggest that progression of ALS may be driven, at least partly, by a self-perpetuating cycle of inflammatory neurotoxicity. In this cycle, intracellular aggressive mSOD1 induces NLRP3 inflammasome-caspase-1 pathway activation in motor neurons firstly, and release of pro-inflammatory IL-1β from neurons, which in turn activates astrocytes and microglia to induce chronic neuroinflammation.

The emerging concept of reactive astrocyte or microglia heterogeneity receives more and more attention. Activated astrocytes and microglia display either beneficial or detrimental actions in context with disease stage and brain (Additional file 7) region during chronic or acute CNS insult [35, 36]. In ALS, discrete subtypes of glial cells with specific molecular and functional properties need to be fully explored.

## Conclusion

**Fig. 9:**
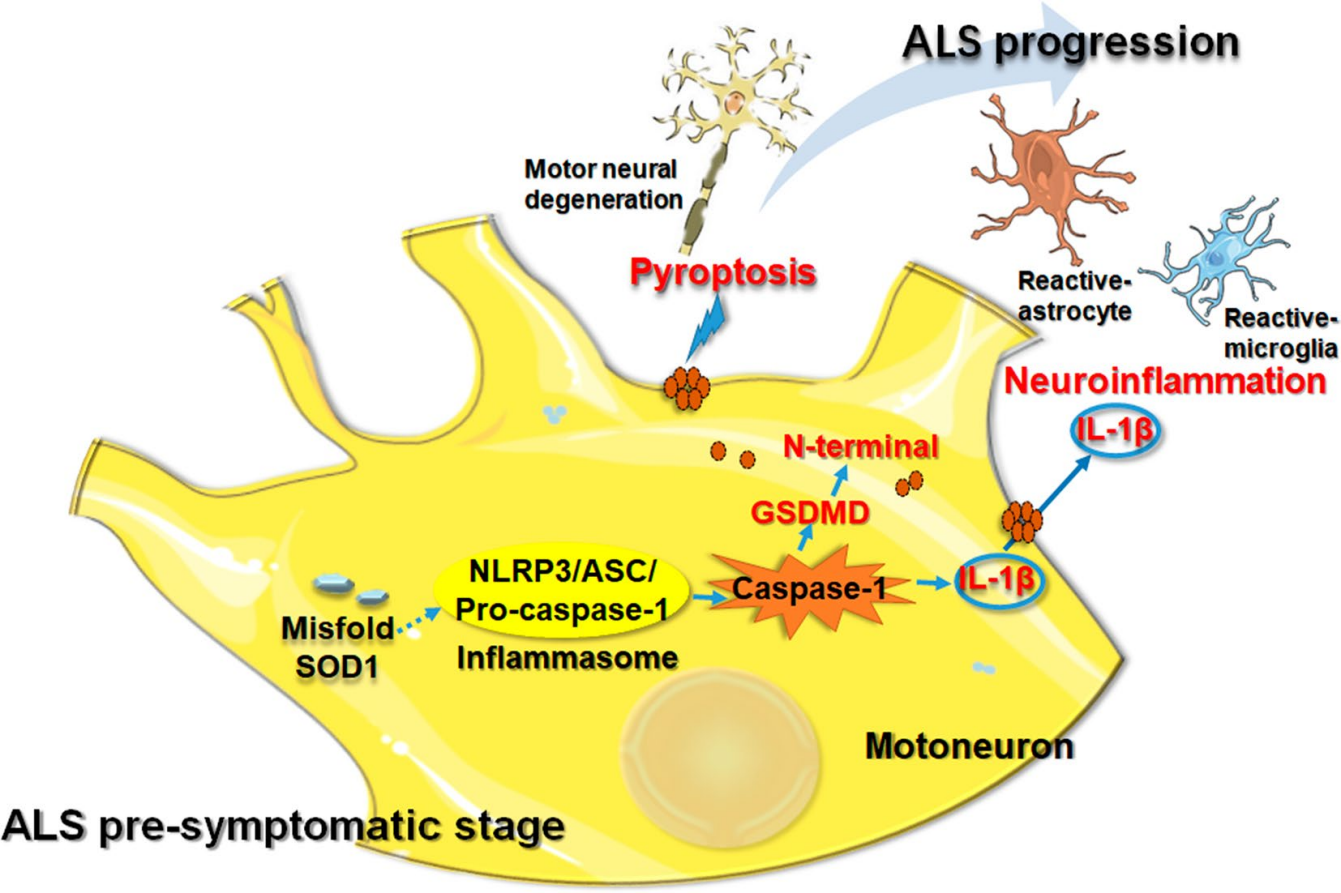
Schematic presentation of NLRP3 inflammasome activation-induced pyroptosis and neuroinflammation in pre-symptomatic stage during ALS progression. From pre-symptomatic stage of ALS, cellular aberrant mSOD1 participates in assemble of NLRP3 inflammasome, which in turn activates pro-caspase-1. Active caspase-1 not only promotes the maturation of IL-1β, but also cleaves GSDMD, releasing its N-terminal fragment. Then these N-terminal fragment translocate to the plasma membrane causing pore formation and inducing pyroptosis. IL-1β released by degenerated motor neurons leads to continuously activate of astrocytes and microglia, which aggravates neuroinflammation, and participates in the progression of ALS

Our study demonstrated the cellular distribution characteristics of pyroptosis and canonical inflammasome in ALS lumbar spinal cord, which were first observed in ventral horn neurons from early stage of ALS. This early activation of the NLRP3 inflammasome and pyroptosis could be involved in motor neurodegeneration and ALS disease progression (Fig. 9). Treatment options targeting the NLRP3 inflammasome might be useful. However, the functional changes of reactive glial cells need to be clarified.

## Supplementary Information

**Additional file 1.** Raw western blots for GSDMD, IL-1β and GAPDH in Figure 2b and 7b.

**Additional file 2.** Magnification images for GSDMD in Figure 3.

**Additional file 3.** Raw western blots for NLRP3 and GAPDH in Figure 4b.

**Additional file 4.** Raw western blots for caspase-1 and GAPDH in Figure 4d.

**Additional file 5.** Magnification images for NLRP3 in Figure 5.

**Additional file 6.** Magnification images for caspase-1 in Figure 6.

**Additional file 7.** Magnification images for IL-1β in Figure 8.

## Data Availability

All data generated or analysed during this study are included in this published article.

## Abbreviations

ALS: Amyotrophic lateral sclerosis
ASC: Apoptosis-associated speck-like protein
CNS: Central nervous system
fALS: Familial ALS
GSDMD: Gasdermin-D
IL-1β: Interleukin-1β
MN: Motor neuron
NLRP: NOD-like receptor protein
NOD: Nucleotide-binding oligomerization domain
qRT-PCR: Quantitative real-time PCR
sALS: Sporadic ALS
SOD1: Superoxide dismutase-1
