# Supplementary material for: Spatiotemporal evolution of pyroptosis and canonical inflammasome pathway in hSOD1G93A ALS mouse model
Source: BMC Neurosci. 2022 Aug 9;23:50. doi: 10.1186/s12868-022-00733-9 (PMC9364624; doi:10.1186/s12868-022-00733-9)
Supplement: Supplementary file 1 — Additional file 1. Raw western blots for GSDMD, IL-1β and GAPDH in Figure 2b and 7b. [file 12868_2022_733_MOESM1_ESM.pptx]

## Slide 1
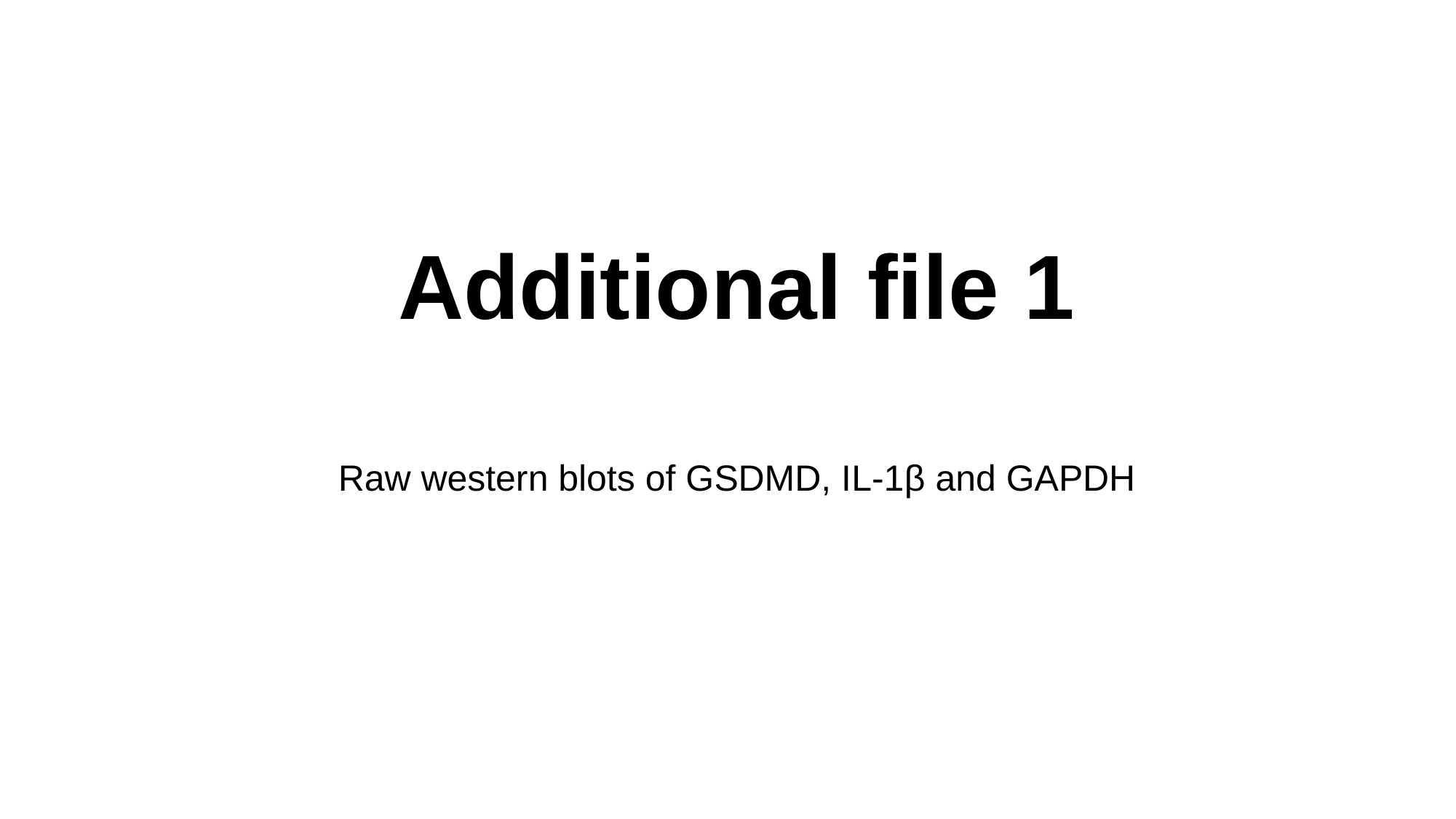

# Additional file 1
Raw western blots of GSDMD, IL-1β and GAPDH

## Slide 2
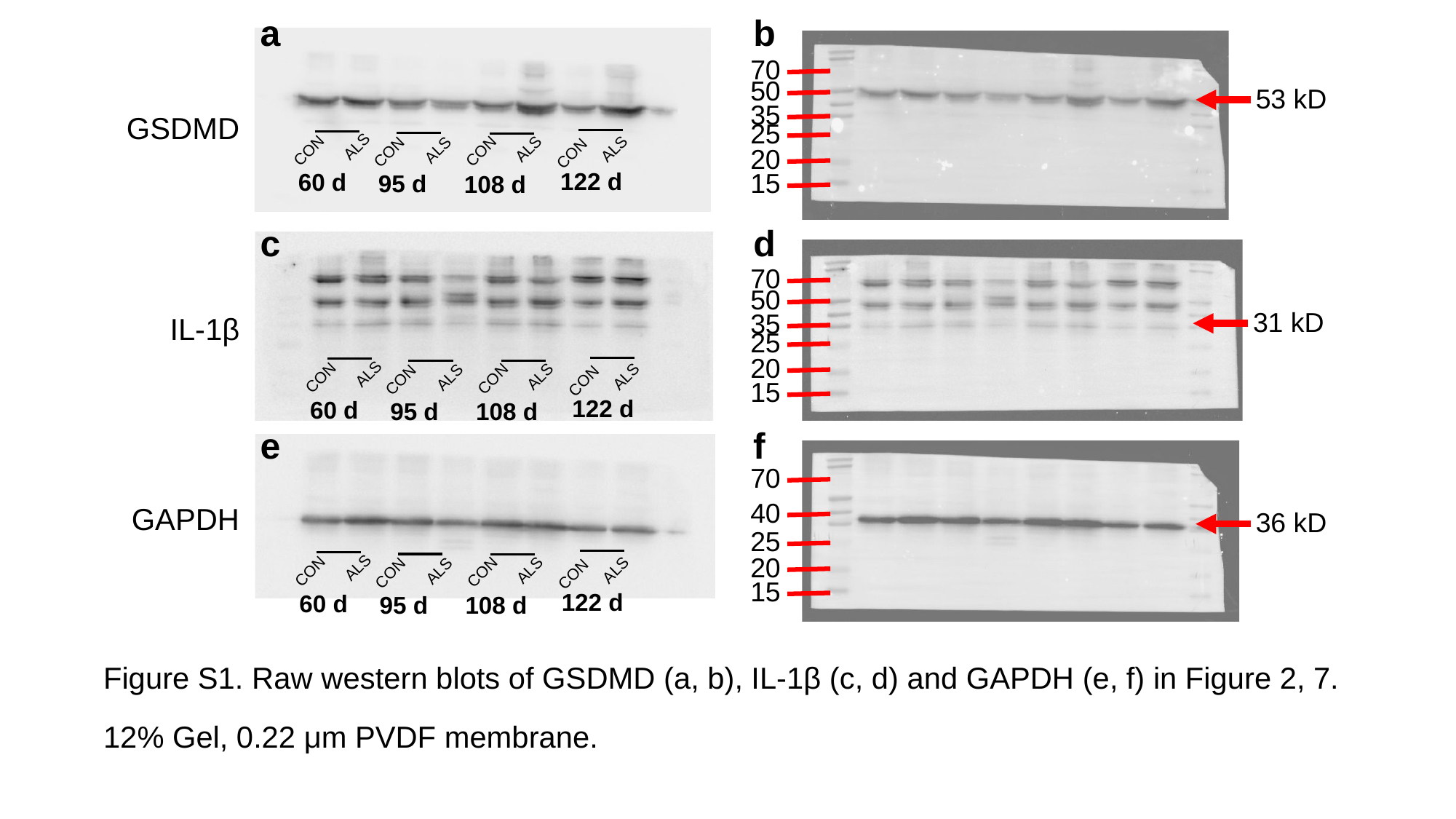

a
b
70
50
53 kD
35
GSDMD
25
ALS
CON
60 d
ALS
CON
95 d
ALS
CON
122 d
ALS
CON
108 d
20
15
c
d
70
50
31 kD
35
IL-1β
25
20
ALS
CON
60 d
ALS
CON
95 d
ALS
CON
122 d
ALS
CON
108 d
15
e
f
70
40
GAPDH
36 kD
25
20
ALS
CON
60 d
ALS
CON
95 d
ALS
CON
122 d
ALS
CON
108 d
15
Figure S1. Raw western blots of GSDMD (a, b), IL-1β (c, d) and GAPDH (e, f) in Figure 2, 7.
12% Gel, 0.22 μm PVDF membrane.
