# Supplementary material for: Spatiotemporal evolution of pyroptosis and canonical inflammasome pathway in hSOD1G93A ALS mouse model
Source: BMC Neurosci. 2022 Aug 9;23:50. doi: 10.1186/s12868-022-00733-9 (PMC9364624; doi:10.1186/s12868-022-00733-9)
Supplement: Supplementary file 3 — Additional file 3. Raw western blots for NLRP3 and GAPDH in Figure 4b. [file 12868_2022_733_MOESM3_ESM.pptx]

## Slide 1
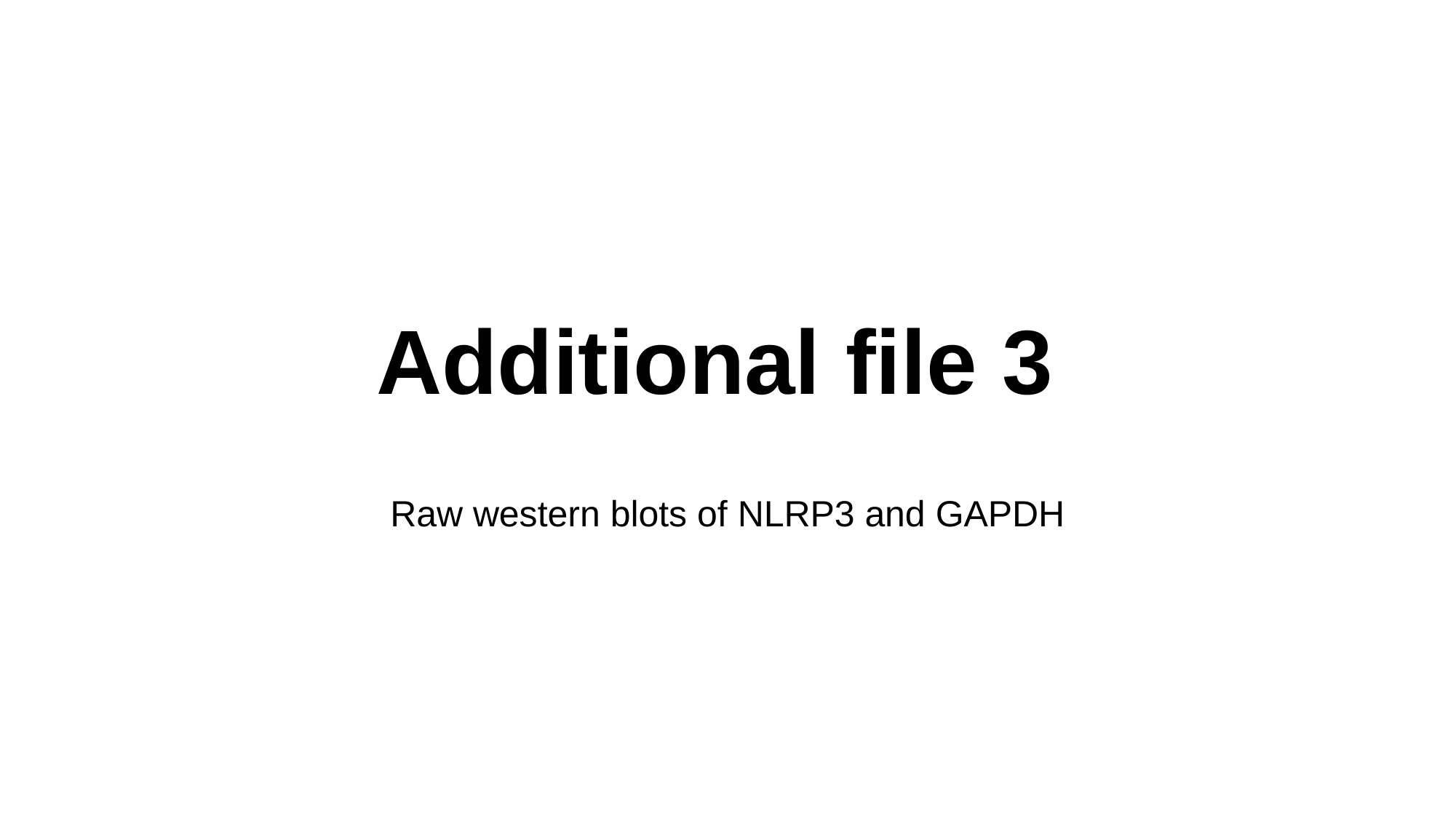

# Additional file 3
Raw western blots of NLRP3 and GAPDH

## Slide 2
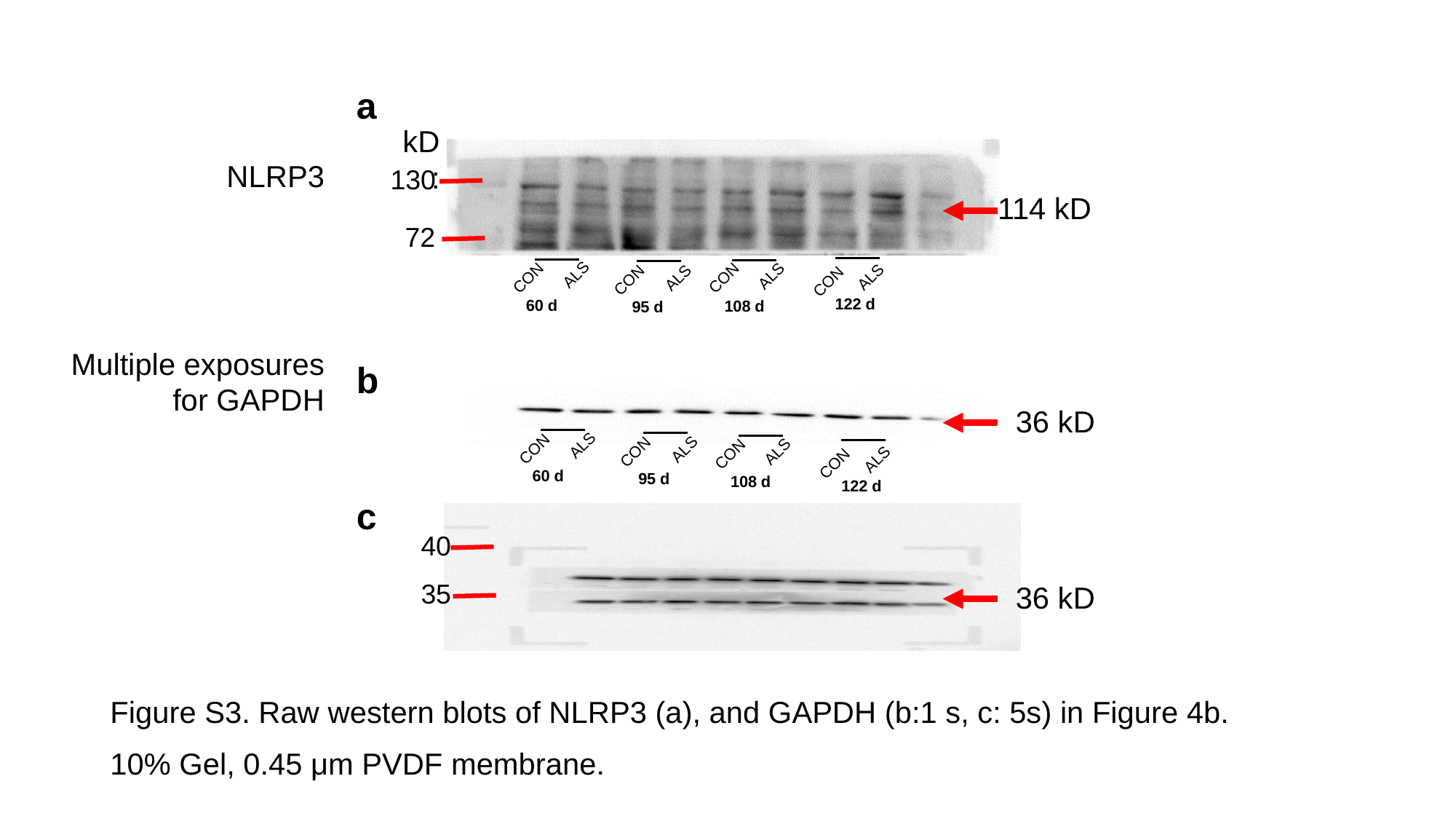

a
kD:
NLRP3
130
114 kD
72
ALS
CON
108 d
ALS
CON
95 d
ALS
CON
60 d
ALS
CON
122 d
Multiple exposures for GAPDH
b
36 kD
ALS
CON
60 d
ALS
CON
95 d
ALS
CON
108 d
ALS
CON
122 d
c
40
35
36 kD
Figure S3. Raw western blots of NLRP3 (a), and GAPDH (b:1 s, c: 5s) in Figure 4b.
10% Gel, 0.45 μm PVDF membrane.

## Slide 3
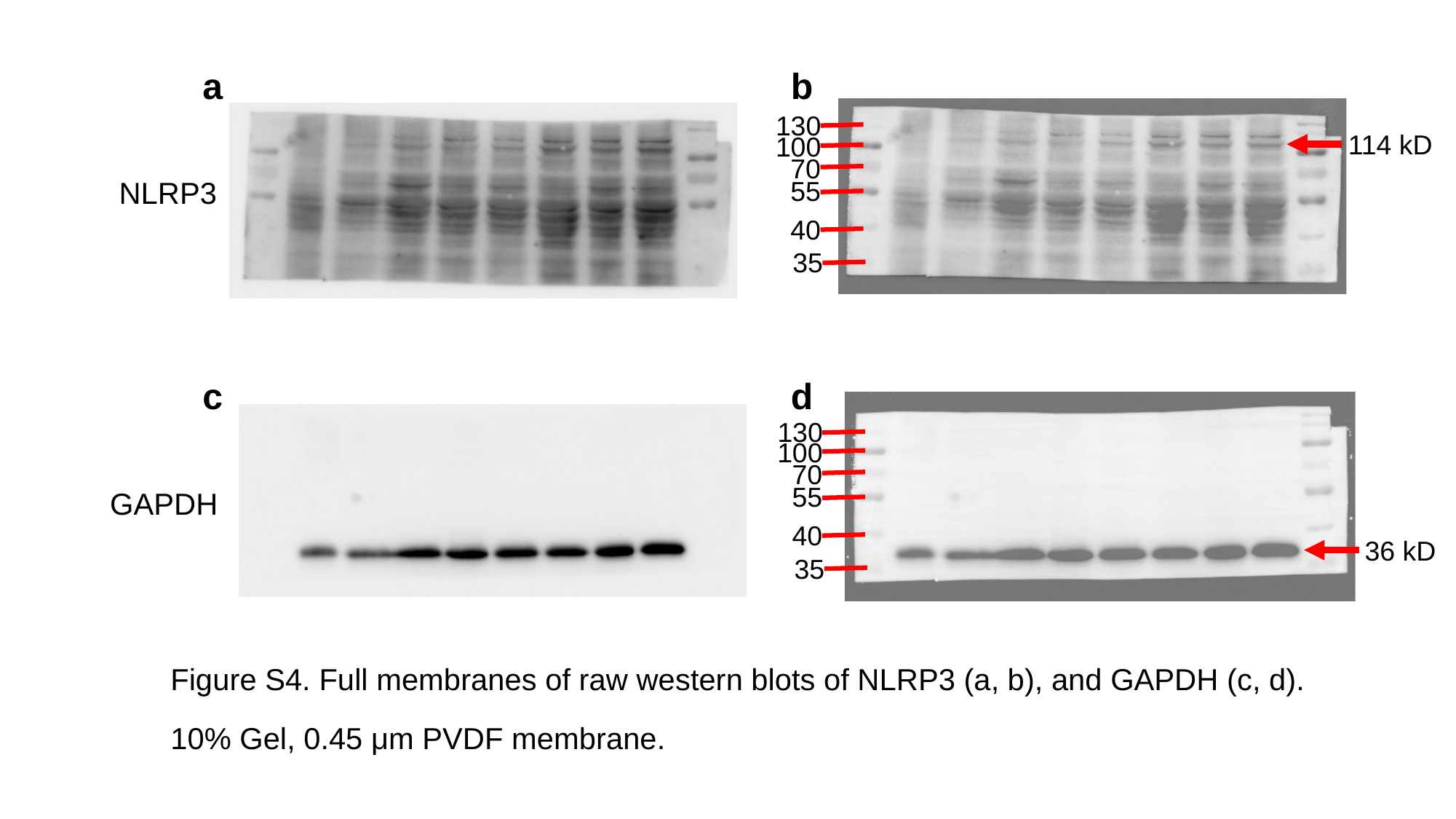

a
b
130
100
70
55
40
35
114 kD
NLRP3
c
d
130
100
70
55
40
35
GAPDH
36 kD
Figure S4. Full membranes of raw western blots of NLRP3 (a, b), and GAPDH (c, d).
10% Gel, 0.45 μm PVDF membrane.
