# Supplementary material for: Spatiotemporal evolution of pyroptosis and canonical inflammasome pathway in hSOD1G93A ALS mouse model
Source: BMC Neurosci. 2022 Aug 9;23:50. doi: 10.1186/s12868-022-00733-9 (PMC9364624; doi:10.1186/s12868-022-00733-9)
Supplement: Supplementary file 4 — Additional file 4. Raw western blots for caspase-1 and GAPDH in Figure 4d. [file 12868_2022_733_MOESM4_ESM.pptx]

## Slide 1
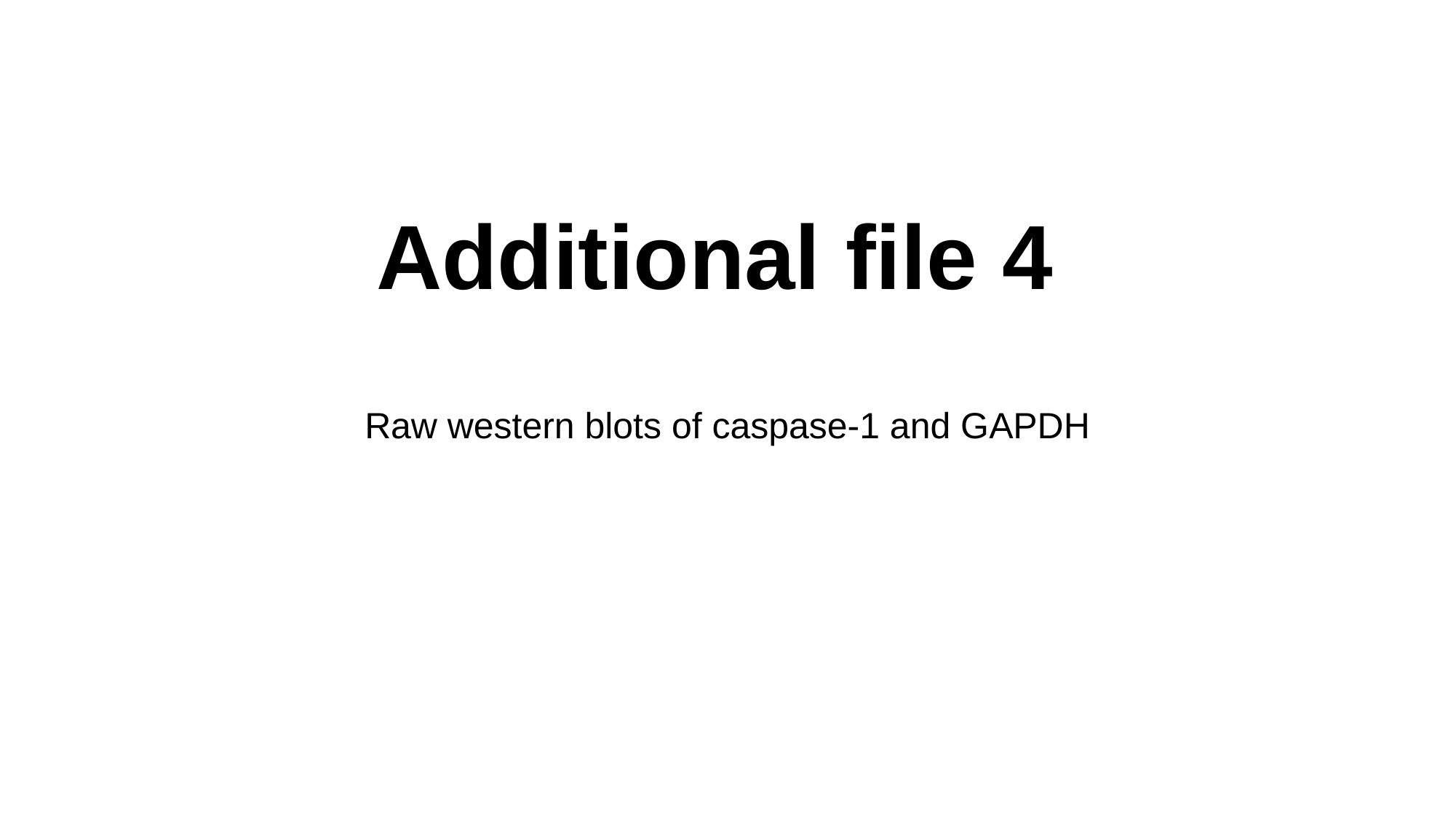

# Additional file 4
Raw western blots of caspase-1 and GAPDH

## Slide 2
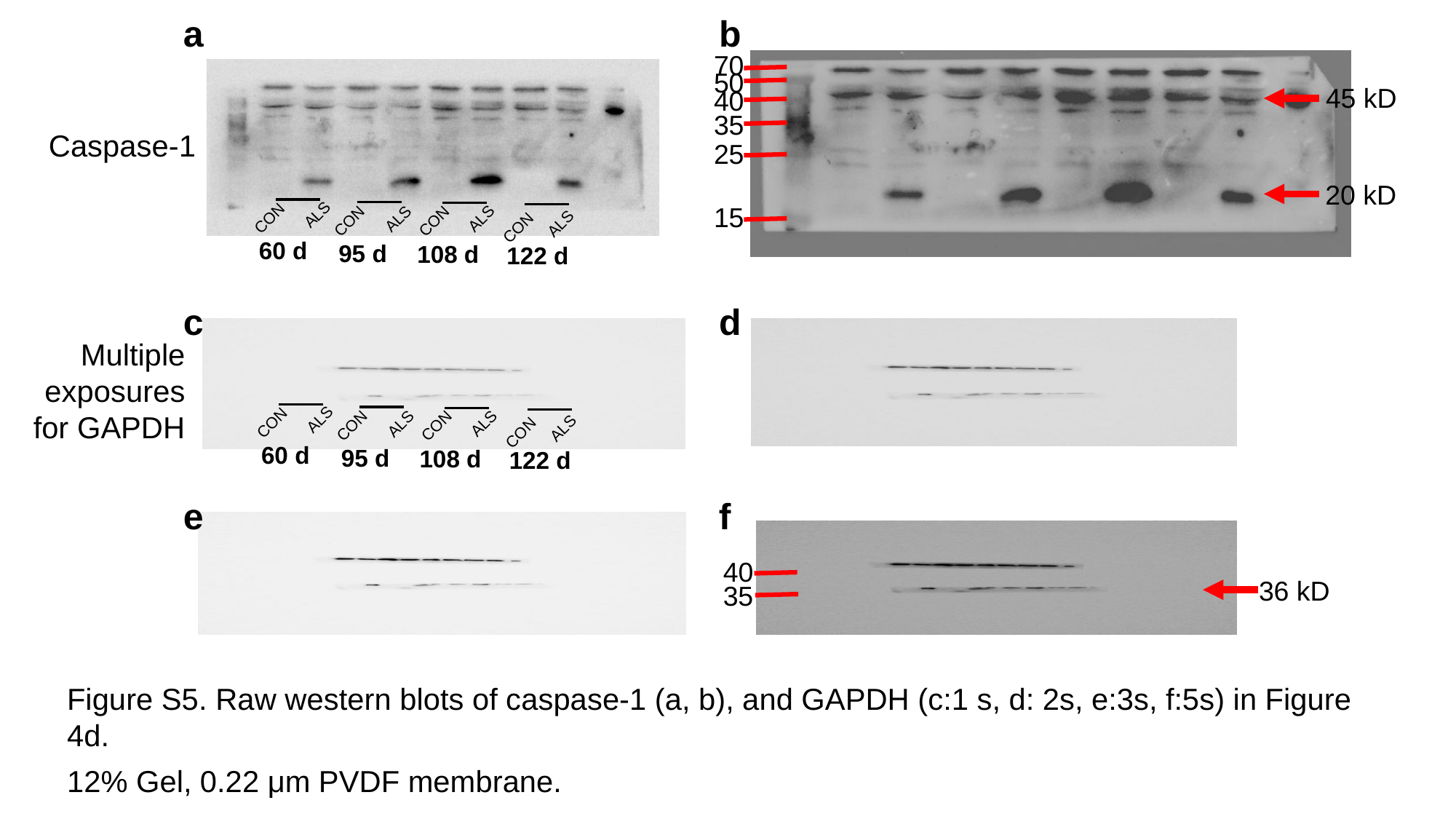

a
b
70
50
45 kD
20 kD
40
35
Caspase-1
25
15
ALS
CON
60 d
ALS
CON
95 d
ALS
CON
108 d
ALS
CON
122 d
c
d
Multiple exposures for GAPDH
ALS
CON
60 d
ALS
CON
95 d
ALS
CON
108 d
ALS
CON
122 d
e
f
40
36 kD
35
Figure S5. Raw western blots of caspase-1 (a, b), and GAPDH (c:1 s, d: 2s, e:3s, f:5s) in Figure 4d.
12% Gel, 0.22 μm PVDF membrane.

## Slide 3
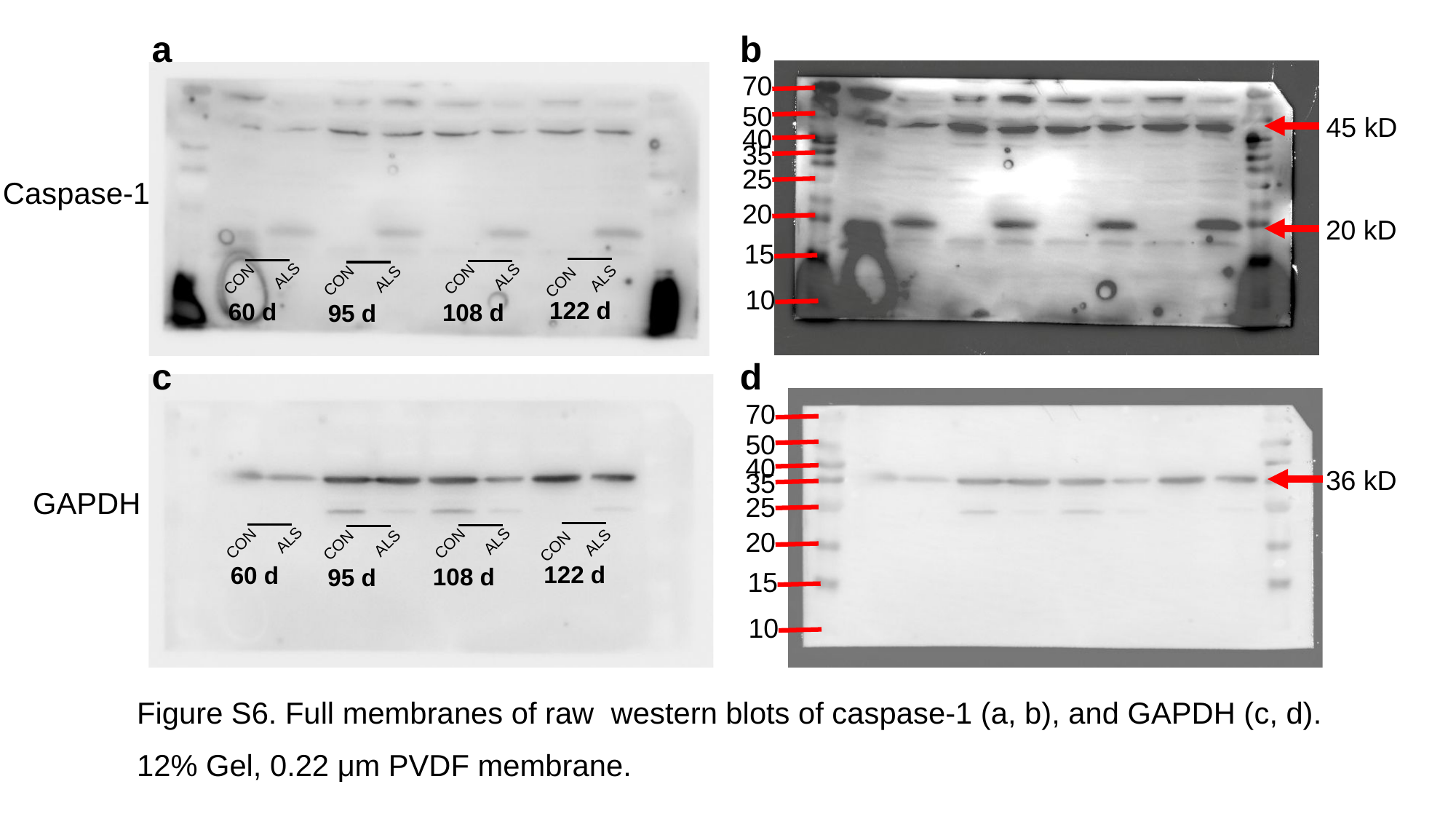

a
b
70
50
40
35
25
20
15
10
45 kD
20 kD
Caspase-1
ALS
CON
60 d
ALS
CON
95 d
ALS
CON
108 d
ALS
CON
122 d
c
d
70
50
40
35
25
20
15
10
36 kD
GAPDH
ALS
CON
60 d
ALS
CON
95 d
ALS
CON
108 d
ALS
CON
122 d
Figure S6. Full membranes of raw western blots of caspase-1 (a, b), and GAPDH (c, d).
12% Gel, 0.22 μm PVDF membrane.
