# Supplementary material for: Spatiotemporal evolution of pyroptosis and canonical inflammasome pathway in hSOD1G93A ALS mouse model
Source: BMC Neurosci. 2022 Aug 9;23:50. doi: 10.1186/s12868-022-00733-9 (PMC9364624; doi:10.1186/s12868-022-00733-9)
Supplement: Supplementary file 7 — Additional file 7. Magnification images for IL-1β in Figure 8. [file 12868_2022_733_MOESM7_ESM.pptx]

## Slide 1
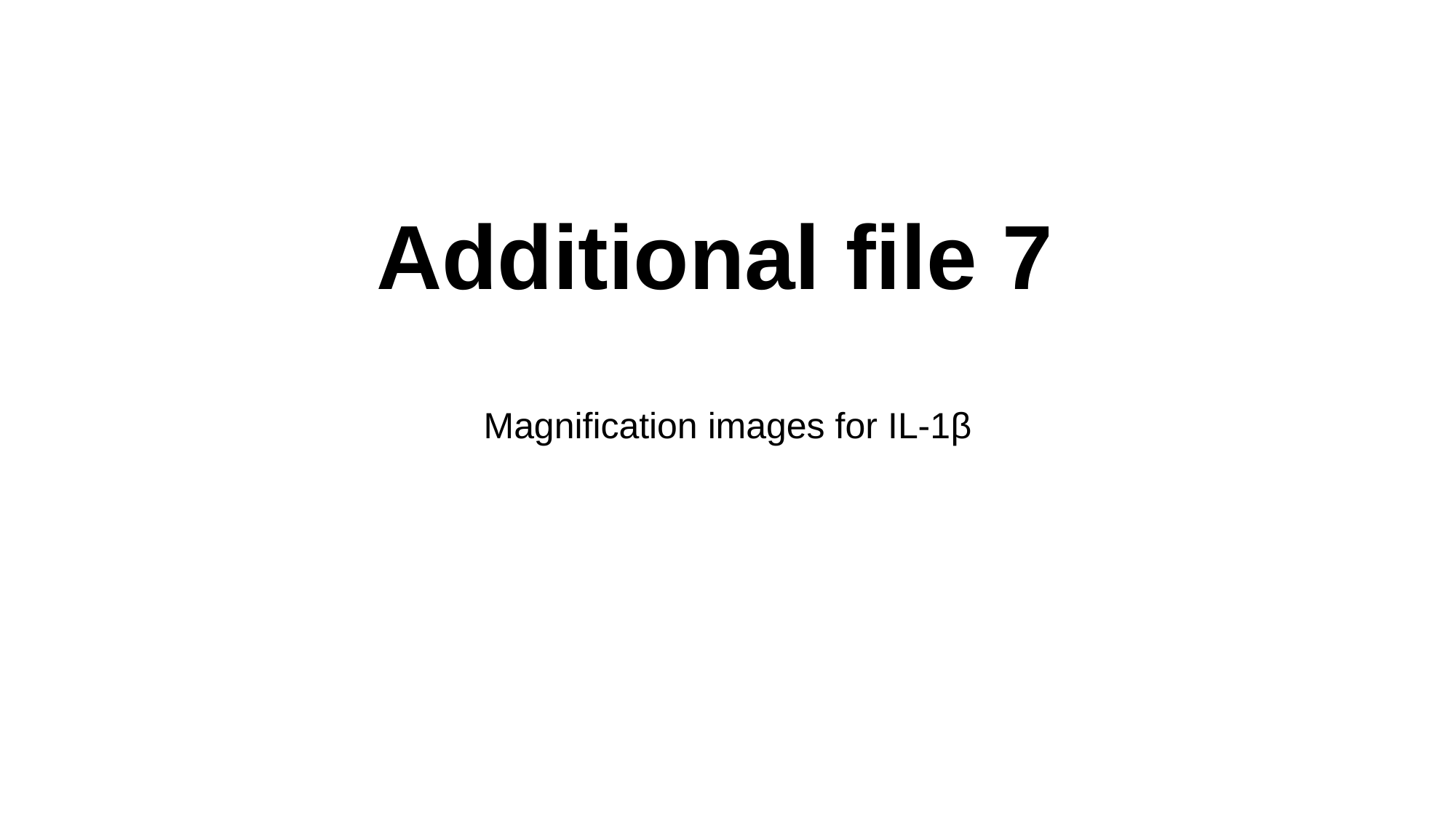

# Additional file 7
Magnification images for IL-1β

## Slide 2
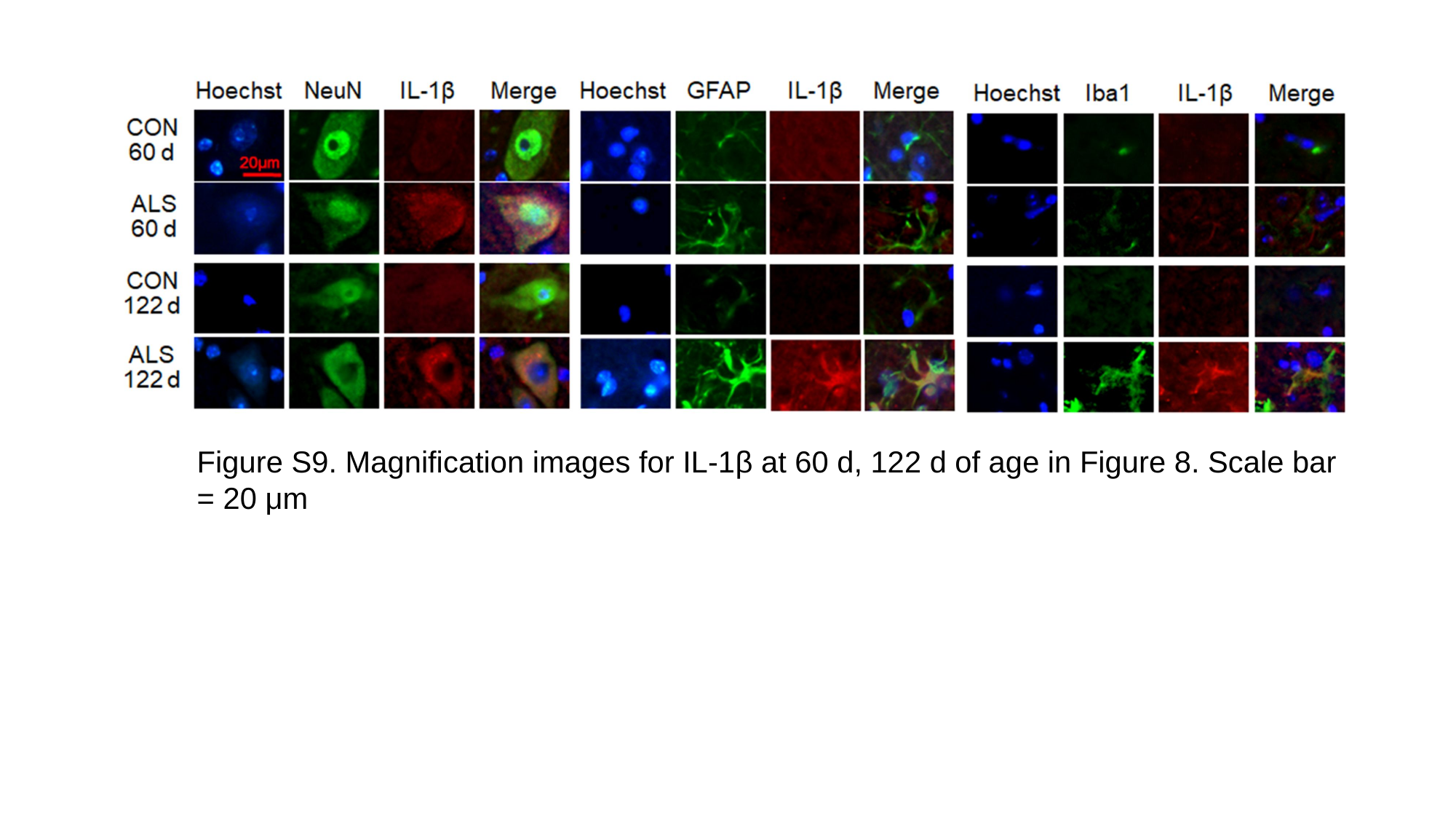

Figure S9. Magnification images for IL-1β at 60 d, 122 d of age in Figure 8. Scale bar = 20 μm
